# Neuroprotection for Stroke: Current Status and Future Perspectives

**DOI:** 10.3390/ijms130911753

**Published:** 2012-09-18

**Authors:** Jens Minnerup, Brad A. Sutherland, Alastair M. Buchan, Christoph Kleinschnitz

**Affiliations:** 1 Department of Neurology, University of Münster, Albert-Schweitzer-Campus 1, 48149 Münster, Germany; 2 Institute of Epidemiology and Social Medicine, University of Münster, Münster 48149, Germany; 3 Acute Stroke Programme, Nuffield Department of Clinical Medicine, University of Oxford, Oxford 38655, UK; E-Mails: brad.sutherland@ndm.ox.ac.uk (B.A.S.); alastair.buchan@medsci.ox.ac.uk (A.M.B.); 4 University Clinic of Würzburg, Department of Neurology, Würzburg 97080, Germany; E-Mail: christoph.kleinschnitz@mail.uni-wuerzburg.de

**Keywords:** neuroprotection, ischemic stroke, translation, STAIR, ischemic cascade

## Abstract

Neuroprotection aims to prevent salvageable neurons from dying. Despite showing efficacy in experimental stroke studies, the concept of neuroprotection has failed in clinical trials. Reasons for the translational difficulties include a lack of methodological agreement between preclinical and clinical studies and the heterogeneity of stroke in humans compared to homogeneous strokes in animal models. Even when the international recommendations for preclinical stroke research, the Stroke Academic Industry Roundtable (STAIR) criteria, were followed, we have still seen limited success in the clinic, examples being NXY-059 and haematopoietic growth factors which fulfilled nearly all the STAIR criteria. However, there are a number of neuroprotective treatments under investigation in clinical trials such as hypothermia and ebselen. Moreover, promising neuroprotective treatments based on a deeper understanding of the complex pathophysiology of ischemic stroke such as inhibitors of NADPH oxidases and PSD-95 are currently evaluated in preclinical studies. Further concepts to improve translation include the investigation of neuroprotectants in multicenter preclinical Phase III-type studies, improved animal models, and close alignment between clinical trial and preclinical methodologies. Future successful translation will require both new concepts for preclinical testing and innovative approaches based on mechanistic insights into the ischemic cascade.

## 1. Introduction

Ischemic stroke, caused by a clot occluding a blood vessel leading to or within the brain, results in impaired blood flow, which produces neuronal cell death. Ischemic stroke is the third greatest cause of mortality and the leading cause of disability in the western world. With an ageing population, putative treatments for ischemic stroke are required to limit the extent of stroke-induced morbidity and mortality. Unfortunately, only one pharmacological agent is routinely administered for acute ischemic stroke, recombinant tissue plasminogen activator (rtPA), a thrombolytic that restores blood flow to the ischemic brain [1]. Currently only 2%–5% of cases receive rtPA treatment (though this figure may be higher in some centers) due to strict eligibility criteria such as time since stroke onset [2]. While the restoration of blood flow by thrombolysis and subsequent delivery of oxygen and nutrients to the ischemic brain is a viable but restricted method to limit neuronal death, targeting the brain parenchyma with pharmacological compounds (called neuroprotection) may also be a promising strategy to curb the spread of infarcted tissue.

The center of the brain region with diminished blood flow following cerebral ischemia is the ischemic core. In this region, blood flow is depleted and neurons undergo irreversible death within minutes. Surrounding the core is the penumbra [3], a region that has reduced blood flow but neurons remain quiescent due to a collateral blood supply. If flow is not restored to the penumbra, these neurons will eventually die and become part of the ischemic infarct. It is the neurons in the penumbral region that neuroprotective agents are attempting to protect.

Neuroprotection is specifically defined as the “protection of neurons” and is a strategy used to potentially protect the brain in a number of different cerebral conditions including Parkinson’s disease, traumatic brain injury and ischemic stroke [4–6]. While pharmacological agents that can prevent clot formation such as anti-thrombotics or antiplatelets, or break down existing clots such as thrombolytics, can produce neuroprotection, these agents primarily target the cerebral vasculature and so are considered extrinsic or indirect neuroprotectants and are not considered further in this article [7]. Agents that directly act upon the neuron itself are considered direct neuroprotectants. Following cerebral ischemia, a complicated cascade of biochemical events occurs, ultimately leading to the death of neurons (see Figure 1 of [6]). Within this cascade, many molecular targets can be pharmacologically modulated to produce neuroprotection [8]. Some of the molecular events that can be targeted by neuroprotectants include amongst others: glutamate release, glutamate receptor activation, excitotoxicity, Ca^2+^ influx into cells, mitochondrial dysfunction, activation of many intracellular enzymes, free radical production, nitric oxide production, apoptosis, and inflammation [6]. In pre-clinical studies, over 1000 potential neuroprotective therapies have been trialed targeting some of the aforementioned molecular events, with many of these treatments providing protection [9]. Unfortunately, after nearly 200 clinical trials, all attempts at neuroprotection for ischemic stroke clinically have failed [10].

In pre-clinical studies, many different methods are used to assess neuroprotection. The predominant method is usually estimating the size of the cerebral infarction volume using histology or magnetic resonance imaging following a model of ischemic stroke such as middle cerebral artery occlusion (MCAO). In light of the fact that assessment of neurological outcome is the primary endpoint in clinical stroke studies, recent trends have been to use functional or behavioral indices to assess neurological deficit in pre-clinical studies [11]. This conforms to the Stroke Academic Industry Roundtable (STAIR) criteria for translation of pre-clinical neuroprotection to clinical trials for ischemic stroke [12,13]. Therefore, for neuroprotection to be achieved pre-clinically, a reduction in infarct volume as well as functional benefit must occur before an agent could be considered for clinical trials. Unfortunately, measuring functional benefit in animal models of stroke is difficult due to the lack of standardization and subjective nature of functional/behavioral testing, as well as lack of correlation with higher brain functioning in humans. Like clinical studies, improving the standardization and validity of functional/behavioral testing for rodents in pre-clinical studies would remove the subjective nature of these tests and enhance the translation of a neuroprotective agent.

As shown by the comprehensive review of neuroprotective compounds by O’Collins *et al.* [9], there is significant variability in the types of compounds tested, and the scale of neuroprotection achieved in pre-clinical animal models. This variability can be attributed to the low methodological quality of many neuroprotective studies [9], based on the STAIR criteria [12,13]. It is clear that many of the compounds that were taken forward into clinical trials had not undergone adequate pre-clinical testing, and so were very unlikely to succeed in clinical trials. The lack of translational success of any neuroprotectant could be due to a number of reasons but many of these are methodological and still do not provide us with a complete picture as to whether a particular compound could fulfill its potential of providing a neuroprotective effect for ischemic stroke in the clinic. Some differences between pre-clinical studies and clinical trials in assessing efficacy for neuroprotective agents have been summarized previously [6], but include: population type (animals are a young, homogeneous population with no comorbidities, while humans who suffer ischemic stroke are usually an elderly, heterogeneous population with numerous comorbidities); ischemic territory (animals are usually restricted to the MCA territory while humans are not); scope for optimization (animal studies have scope for optimizing therapeutic time window, dose, and route of administration while clinical studies do not); occlusion duration (animal studies have controlled duration of occlusion while in humans, occlusion duration is variable); primary endpoint (animal studies use infarct volume, while human studies use functional testing). In addition, confounding physiological effects such as temperature and blood flow need to be closely monitored to assess if an agent is producing neuroprotection by modulating these parameters [14]. These differences between animal and human studies are now being considered when designing pre-clinical studies. More stroke research labs are using older animals and animals with co-morbidities such as diabetes and hypertension, as well as functional testing for neurological deficit as described above. These advances will more closely align pre-clinical studies to clinical trials and it is hoped that they will improve the chances of successful translation for neuroprotection.

Neuroprotection for ischemic stroke from a translational standpoint has recently been reviewed [6]. The present article attempts to add further insight into neuroprotection by highlighting where neuroprotection research is at experimentally and clinically, explaining why previous attempts have failed and highlighting some promising potential neuroprotectants that are in development.

## 2. The Current Status of Experimental and Clinical Neuroprotection Research

The process of developing new neuroprotective stroke treatments usually progresses from preclinical to clinical studies. In animal models a treatment’s mechanisms of action and its efficacy regarding infarct size reduction and functional outcome are investigated. As described above, numerous potential targets for neuroprotective strategies for stroke were identified including inflammation, neuronal apoptosis, free radical damage, excitotoxicity, and calcium influx into cells. Among these impeding excitotoxicity was the most targeted mechanism in animal experimental stroke [9]. More than 20 drugs aiming to attenuate excitotoxicity were tested in more than 270 preclinical studies [9]. Overall, in the period covering 1957 to 2003 O’Collins *et al.* identified publications on 1026 candidate stroke drugs of which about two thirds were superior to control treatments [9]. Despite the disappointment that none of these treatments was shown to be beneficial in a clinical trial, the number of experimental studies on candidate neuroprotective drugs even increased over the last few years [7]. Promising experimental therapies still in preclinical development will be discussed later in the article.

Since neuroprotection for ischemic stroke was first studied, there have been nearly 200 clinical trials using potential neuroprotective agents for ischemic stroke. As described earlier, not one treatment has proven to be effective at providing functional benefit to ischemic stroke patients, even though many of these agents had varying mechanisms of action by targeting different aspects of the ischemic cascade. Despite this apparent failure of clinical neuroprotection, there are a number of ongoing clinical trials investigating promising neuroprotectants that have shown pre-clinical efficacy [10], of which we will name a few.

One of the most hopeful neuroprotective strategies that is currently under clinical investigation is hypothermia, the act of cooling the brain temperature down. Hypothermia has shown significant pre-clinical efficacy in animal models of cerebral ischemia [15], and has been shown to be feasible in acute stroke patients using either surface cooling [16] or endovascular cooling [16,17]. While there has been no evidence of improvement in clinical outcome with mild therapeutic hypothermia to date [16–18] no prospective large controlled clinical trial has yet been carried out. Interestingly, mild therapeutic hypothermia has been shown to be effective at reducing neurological deficit following cardiac arrest [19]. Proposed mechanisms of the neuroprotective action of hypothermia include preventing formation of free radicals, slowing cellular metabolism, reducing glutamate release and diminishing protein kinase C activity [20–22]. Hypothermia can lead to a number of complications including shivering (which can be treated with anti-shivering agents such as buspirone), pneumonia, infections, hypotension, cardiac arrhythmias, and an increase in intracranial pressure during the rewarming period [17,23–25] but these appear to not have been an issue in the few small clinical studies published so far [16,17,25]. Even with the lack of efficacy from the initial feasibility trials [16–18] and associated complications, hypothermia remains a potentially viable strategy for neuroprotective therapy for ischemic stroke and is currently being investigated in both safety and efficacy trials—Cooling in Acute Stroke (COAST-II) trial, Controlled Hypothermia in Large Infarction (CHILI) trial, and the European Stroke Research Network for Hypothermia (EuroHYP)-1 trial [10,26].

Despite the failure of NXY-059 (discussed later in the article), two free radical scavengers are currently being investigated in clinical trials for neuroprotection for stroke. Ebselen, a glutathione-peroxidase mimic reduced infarct volume by 27% on average in nine focal ischemia studies with 10 of the 16 experimental contrasts showing protection [9]. The differences in experimental design and other factors may contribute to why some studies showed protection while other studies revealed no effect of ebselen. A subsequent study has shown that ebselen provided protection against delayed neuronal death and oxidative damage from focal ischemia in hypertensive rats when administered 24 h post-MCAO [27]. Ebselen has also shown protection in transient forebrain ischemia [28,29], and initial clinical studies showed some promise in patients with acute ischemic stroke [30]. A phase III trial exploring the efficacy of ebselen in patients with a cortical infarct is currently ongoing [10]. In contrast with ebselen, edaravone, a hydroxyl radical scavenger, showed poor pre-clinical efficacy with a meta-analysis revealing it worsened outcome in focal rodent models [9]. Despite this, it has shown promise in some clinical trials for ischemic stroke [31,32], and is still undergoing investigation in further clinical trials [10].

Other selected neuroprotective strategies that are currently undergoing investigation include magnesium sulphate, statins, DP-b99, minocycline, and albumin. Magnesium produced significant protection in preclinical animal models of stroke [33] by inhibiting the NMDA receptor and excitotoxicity [34], and inducing hypothermia [16,26]. A pilot clinical trial showed that administration of magnesium sulphate to acute stroke patients by paramedics before arrival at the hospital was safe and associated with a beneficial outcome [35], which has led to a large phase III clinical trial [10]. However, results of the large MASH-2 trial, which showed that magnesium is not superior to placebo regarding the clinical outcome after subarachnoid hemorrhage, put a damper on the hopes for magnesium as therapy for cerebrovascular diseases [36].

Statins block the rate-limiting enzyme of cholesterol production hydroxymethylglutaryl-coenzyme A (HMG-CoA) reductase, but also improve blood flow through increased nitric oxide availability as well as anti-inflammatory and antioxidant effects [37]. Now, a phase II trial with lovastatin for stroke is ongoing [10], which was initiated following promising pre-clinical [9,38] and clinical studies [39]. Another avenue for neuroprotection is scavenging divalent metal ions such as calcium and zinc that are involved in the ischemic cascade leading to cell death [40]. Use of DP-b99, a divalent metal ion chelator produced neuroprotection pre-clinically for cerebral ischemia [40] and was associated with improvement in a Phase II trial for acute ischemic stroke [41], which has initiated a Phase III trial [42] that is currently suspended [43]. Minocycline, an antibiotic, produced neuroprotection preclinically [33] through anti-inflammatory and anti-apoptotic effects [44], and prevented infections following immunosuppression from stroke [45]. Clinical acute stroke studies have shown promising effects with minocycline [45–47] and so is undergoing a Phase III clinical trial [10]. Albumin prevents the leakage of fluid from the vasculature into the tissue [48] which may be useful following cerebral ischemia by attenuating swelling and enhancing blood flow [7]. Pre-clinically, albumin has shown significant neuroprotection [49], while in clinical studies albumin treatment was associated with a favorable outcome in acute stroke patients [50–52]. The results of a phase III trial of albumin therapy in acute stroke are eagerly awaited [53].

## 3. Examples and Reasons for the Translational Failure and Future Strategies to Overcome It

As mentioned above, a wide variety of candidate neuroprotectants was shown to be beneficial in preclinical studies, whereas none were demonstrated to be superior to placebo in clinical trials [9]. Here we describe two case examples of treatments, NXY-059 and haematopoietic growth factors, that were assumed to be particularly promising regarding their clinical potential.

Free radicals have long been established as a mediator of ischemic injury [54]. Many nitrones including disodium 2,4-disulfophenyl-*N*-*tert*-butylnitrone (NXY-059) act as a free radical trap that could potentially inhibit ischemic cell death [55]. Many pre-clinical studies have shown neuroprotection following cerebral ischemia with NXY-059 [56], with a meta-analysis suggesting it reduces infarct volume by as much as 43% [57]. To mimic the human condition more closely, NXY-059 was also protective in a stroke model in non-human primates (marmosets) [58]. NXY-059 fulfilled all of the STAIR criteria and posed an attractive therapy for neuroprotection in human ischemic stroke. The first clinical trial SAINT I [59] produced excitement amongst the stroke community since NXY-059 improved disability significantly 3 months following stroke. In addition, patients who received NXY-059 in conjunction with thrombolysis had reduced hemorrhagic transformation compared to patients receiving thrombolysis alone [59]. However, these results could not be replicated in the statistically more powerful SAINT II trial [60] and a pooled analysis of the two trials revealed no functional benefit of NXY-059 [61]. These results proved disappointing considering the substantial pre-clinical efficacy observed with NXY-059.

There have been a number of reasons given as to why NXY-059 efficacy could not be translated to human ischemic stroke. Pre-clinically, it was shown that NXY-059 may cross the BBB in only small quantities suggesting that its action on the neurovascular unit may mediate its protective effect rather than in the brain parenchyma [62,63]. Overall methodological quality for pre-clinical NXY-059 studies was low [58] even though it fulfilled the STAIR criteria [64]. Many studies did not specify whether there was any randomization or blinding [58], while publication bias may have contributed to the inflated neuroprotective effects of NXY-059 [57]. In addition, only 9% of studies confirmed MCA occlusion with CBF [64] and so may have contributed to the lack of infarction in the NXY-059 groups [65]. There were also methodological differences between the pre-clinical studies and the SAINT trials which may have contributed to the translational failure of NXY-059. The maximum time window for efficacy in animal studies was 4 h post-ischemia onset, which did not match the enrolment period of 6 h used in the SAINT trials [66]. Also, clinical trials should have initially studied a subset of stroke patients that more closely resembled the MCA occlusions that had shown efficacy in animal experiments, instead of also including patients with lacunar or posterior strokes [66].

The well-known and eponymous function of haematopoietic growth factors is to regulate the mobilization, proliferation, maturation and the survival of bone marrow-derived cells [67]. Recently, actions of haematopoietic growth factors paralleling those in the haematopoietic system were identified in the brain [68,69]. Functions are mediated by haematopoietic growth factor receptors expressed on neurons. Based on results of extensive animal experimental stroke studies erythropoietin (EPO) and the granulocyte-colony-stimulating factor (G-CSF) were thought to be particularly promising for further clinical development. Mechanisms of action of EPO and G-CSF include the reduction of glutamate-induced neuronal cell death and anti-inflammatory effects after cerebral ischemia [69–72]. Beyond their neuroprotective properties, G-CSF and EPO improve post-stroke regeneration by enhancing neurogenesis and angiogenesis [69,73,74]. The large number of animal stroke studies on EPO and G-CSF enabled preclinical meta-analyses which convincingly demonstrated that both factors reduce infarct volumes and improve functional outcomes [75–78].

In a pilot clinical trial EPO was safe in stroke patients and there was some evidence that EPO might improve outcome [79]. These initial results, however, could not be confirmed by a subsequent larger study [80]. Besides missed primary endpoints on efficacy, the mortality rate was even increased after EPO treatment. The differences between results of preclinical and clinical studies might be explained by an overestimated efficacy of EPO in animal experiments through neglected quality characteristics [77,81]. Moreover, the higher mortality after EPO treatment in the clinical study might be due to human specific side effects which could not be anticipated in animal studies [81].

A phase IIa clinical trial showed that G-CSF was safe in stroke patients even at high doses [82]. In this trial dose-dependent beneficial effects of G-CSF were demonstrated for patients with large infarcts. However, a larger study (AXIS-2) of 328 stroke patients receiving either G-CSF or placebo within 9 h after stroke onset found no differences regarding the primary endpoint, clinical outcome at day 90 [83]. So far, the study is only published as an abstract. Potential explanations for the failed translation of G-CSF are expected when the study is published as a full paper.

The two case examples of failed neuroprotective treatments for stroke patients outlined the problems associated with translation. Reasons for translational difficulties have been discussed exhaustively but include variable outcome measures, clinical trial design flaws, delayed treatment time window, small sample sizes, and failure to achieve sufficient plasma levels of treatments [6,84,85]. In addition, inappropriate animal stroke models may overestimate the efficacy of candidate neuroprotectants. Animals used in stroke studies are usually young and healthy, whereas patients are typically older and have various comorbidities, such as diabetes or hypertension. Moreover, the heterogeneous nature of human stroke is not well reproduced in animal models [86]. Due to their homogeneity animal models mimic at best less than 25% of all strokes [87]. Therefore, new animal models are required that better reflect the heterogeneity of human ischemic stroke. Overall, there is a need for a more rigorous design of animal stroke studies with higher quality standard levels to avoid bias [7,57].

New concepts to improve translation from animal experimental studies to clinic comprise applying the process of clinical drug development to the preclinical situation [88]. This includes a multi-stage approach which progresses from phase I to phase II to phase III pre-clinical studies. Following such a strategy phase I pre-clinical studies would aim to discover or investigate pathophysiological mechanisms of a treatment. Phase II pre-clinical studies evaluate the efficacy of a drug and further investigate its safety by individual scientists. Before moving to clinical trials with a compound that was successfully evaluated in pre-clinical phase I and phase II studies, testing in international, multicenter pre-clinical phase III-type studies is required to confirm its efficacy. Another characteristic of clinical drug development, trial registration in a registry including the a priori definition of primary and secondary endpoints, might be adapted to pre-clinical studies. Comparable to clinical trials, registration would be mandatory for publication of pre-clinical studies. A further approach to reduce the known publication bias, which exists in pre-clinical stroke studies and considerably contributes to the overestimation of a treatment’s efficacy, was followed by some stroke journals by increasing the number of publications containing animal studies with neutral results [89–91].

Other strategies to improve outcome after stroke include treatments that promote neurological recovery by remodeling of brain tissue (for a review see [92]). By contrast with neuroprotection this neurorestorative approach might not necessarily require a circumscribed therapeutic time window. Another potential strategy is to use a combination therapy approach, such as a neuroprotective agent alongside thrombolysis, which could produce synergistic protective effects through different mechanisms. Recombinant tissue rtPA is the only approved thrombolytic agent for acute ischemic stroke and protects the brain by recanalisation of an occluded vessel restoring blood flow to the ischemic brain, whereas neuroprotectants act directly upon the brain. Combination therapy could also potentially extend the current therapeutic time window of rtPA from 4.5 h and reduce adverse effects such as intracerebral hemorrhage. Combining rtPA treatment with neuroprotective agents such as NMDA receptor antagonists [93], free radical scavengers [94,95] and matrix metalloprotease inhibitors [96] has shown synergistic efficacy pre-clinically. However, combination strategies have yet to be thoroughly examined clinically, with the few combination trials showing no additional benefit [60,97,98].

## 4. Promising Neuroprotective Treatments

Despite all the disappointments there are still potential neuroprotective compounds that are currently being investigated for the treatment of acute stroke (Table 1). Here we discuss four groups of neuroprotective agents that have shown promising effects in animal studies.

Free radical formation including ROS (reactive oxygen species) contributes to secondary infarct growth [99–101]. However, besides their detrimental effects ROS also exhibit essential signaling functions such as regulating the vascular tone, oxygen tension and erythropoietin production [102]. Only if the balance of pro- and anti-oxidants is shifted towards an exceeding pro-oxidants production, oxidative stress with its harmful consequences arises. The fact that antioxidants investigated in previous studies did not distinguish between physiological and pathological ROS may have contributed to their disappointing results in clinical trials [103]. Moreover, one has to consider that ROS may also cause damage already before they are inactivated by antioxidants. Future stroke research therefore should tackle the oxidative stress at its root and more specifically at the disease-relevant source of ROS, rather than attempting to detoxify them in an untargeted fashion after they have been formed [104]. Although not assuredly identified so far, NADPH oxidases seem to be the main source of ROS in the ischemic brain. These enzymes, in contrast to others such as xanthine oxidase and cytochrome P450 enzymes, require no initial oxidation step and NADPH oxidases are the only enzymes solely dedicated to ROS production [105]. Initially, NADPH enzymes were characterized regarding their role in the immunological host defense of neutrophils. More recently, it was demonstrated that the catalytic subunit of the phagocytic NADPH-oxidase is only one member of a family of 4 homologous proteins known as NOX1-4 (for NADPH-oxidase). We have shown that NOX4-derived oxidative stress plays a pivotal role in the pathophysiology of cerebral ischemia [106,107]. After human stroke and in animal stroke models NOX4 is massively induced in neurons and brain vessels. Genetic or pharmacologic NOX4 inhibition in stroke mice induces an exceptional neuroprotection resulting in significantly improved long-term neurological function and reduced mortality [107]. In line with these findings, overexpression of NOX4 in cerebral endothelial cells enhanced neuronal cell death upon experimental stroke [108]. Blocking a specific enzymatic source of ROS rather than using unspecific antioxidants after free radicals were already formed seems to be a promising treatment option for acute stroke [109]. NOX inhibitors with improved pharmacological properties, specificity for distinct subtypes, and a fair safety profile will probably be available for clinical trials in the near future [104].

Another promising approach includes the inhibition of the NMDA receptor associated protein PSD-95 (postsynaptic density protein-95). This protein binds both NMDA receptors and neuronal nitric oxide synthase (nNOS) at excitatory synapses and assembles them into a signaling complex [110]. Activation of nNOS with subsequent nitric oxide generation, a mediator of glutamate-mediated excitotoxicity, depends on PSD-95 and on NMDA receptor mediated calcium influx [111]. Inhibition of PSD-95 prevents the formation of the NMDA-receptor/PSD-95/nNOS complex and thereby reduces the production of harmful nitric oxides. Other key physiological functions in the CNS mediated by NMDA receptors are not affected by PSD-95 inhibition [110,112]. This is particularly important; because the failure of previous studies of NMDA receptor antagonists was related to side effects due to the blockade of NMDA associated ion-flux and prosurvival signaling pathways [113]. Indeed, PSD-95 inhibition was shown not to exert NMDA receptor mediated side effects, making this compound a suitable and safe therapy for humans [114]. PSD-95 inhibitors were demonstrated to reduce infarct volumes in a mouse stroke model when given up to three hours after the onset of ischemia [114,115]. In addition to its efficacy in rodents, PSD-95 inhibition exerts neuroprotective properties in gyrencephalic non-human primates [116]. The rigorous testing in higher-order primates with a clinically meaningful therapeutic time window make PSD-95 inhibitors a particularly promising neuroprotectant for stroke treatment [117].

Another attractive target could be the activation and stabilization of hypoxia-inducible factor (HIF). HIF (isoforms 1–3) are activated under conditions of low oxygen tension and stimulate many biological processes such as vascular tone, erythopoiesis and angiogenesis [118]. Under normoxic conditions, the hydroxylation of HIF is catalysed by iron- and 2-oxoglutarate (2-OG)-dependent oxygenases (the main subtype being prolyl-4-hydroxylases (PHD)), which targets HIF for proteasomal degradation [119]. Under ischemic conditions, the hydroxylation of HIF is prevented by the lack of oxygen allowing HIF to activate a number of gene pathways including vascular endothelial growth factor (VEGF), endothelial nitric oxide synthase (eNOS) and EPO [119]. While these pathways have been investigated individually for neuroprotection [78,120,121], activating all of these pathways in concert by stabilizing HIF may lead to a greater chance of neuroprotection following stroke. Inhibiting the PHDs with small molecules stabilizes HIF in the brain [122] and produces neuroprotection in rodent models of cerebral ischemia [122–124]. Furthermore, genetic ablation of the PHD2 isoform led to improved functional benefit following MCA occlusion in mice [125]. However, small molecule inhibitors used to block PHD activity can also inhibit other 2-OG-dependent enzymes which affects many cellular processes including DNA repair and histone methylation [126]. HIF remains an appealing target due to its influence over many signaling pathways crucial for protection, but until specific small molecule inhibitors of the PHDs are produced to limit the off-target effects, the translational potential of this neuroprotective target will remain elusive.

Another potential therapeutic approach is targeting inflammation following ischemic stroke. Cytokines such as interleukin-1 (IL-1) have been implicated in neuronal injury following ischemic stroke as well as traumatic brain injury and excitotoxicity [127]. Exogenous administration of IL-1 exacerbated ischemic injury [128] and so targeting IL-1 provides a promising avenue for neuroprotection against ischemic injury. IL-1 receptor antagonist (IL-1ra) is a naturally occurring competitive antagonist to the IL-1 receptor and so can regulate IL-1’s functions including the inflammatory response [129]. Exogenous administration of IL-1ra out to 3 h following transient MCAO in the rat can protect against ischemic brain injury [130]. Furthermore, a meta-analysis of all pre-clinical ischemia studies revealed that IL-1ra produced a 38% reduction in infarct volume over seventeen studies [131]. Efficacy of IL-1ra was improved when higher doses were used, it was centrally administered and treatment was earlier [131]. IL-1ra has also shown pre-clinical efficacy following transient MCAO in aged rats with comorbidities making its efficacy clinically relevant for human stroke populations [132]. IL-1ra has been shown to cross the blood-brain barrier in both rats [133] and humans [134] at doses which could provide therapeutic benefit. IL-1ra has also been tested in a Phase II clinical stroke trial. Intravenous administration of IL-1ra within 6 h of acute stroke onset was safe and well tolerated, while the inflammatory state and clinical outcome of patients on IL-1ra was improved compared to placebo at 3 months [135]. IL-1ra treatment also reversed peripheral innate immune suppression that is associated with the acute phase of stroke [136]. Overall, IL-1ra can block IL-1 function which has demonstrated efficacy both pre-clinically and clinically. However, a Phase III multi-center clinical trial is required to confirm its therapeutic potential for ischemic stroke.

## 5. Conclusions

The failed translation from animal experimental stroke studies to clinical studies has created a great deal of pessimism over the neuroprotection hypothesis. Although animal stroke research has not directly yielded new clinical drugs, it has provided important mechanistic insights into the complex pathophysiology of ischemic stroke which will pave the way for future therapies. Moreover, we have learnt that the implementation of quality standards in experimental studies is not a panacea, but future neuroprotection experiments should nevertheless adhere to these methodological standards. Suggested multicenter preclinical Phase III-type studies might be a further concept to improve the evaluation of candidate neuroprotectants before moving to clinical trials. However, one has to keep the balance between high quality on the one hand, and practicability for preclinical research groups on the other hand. The validation of results from rodent experiments in higher-order primates sounds reasonable and might close the gap between rodent and human studies. Primate experiments, however, raise ethical concerns considering stroke is such an injuring and disabling condition. Practical concerns exist due to the high costs of primates and only a few primate facilities exist across Europe [137]. Future successful developments of stroke drugs will certainly require both new concepts for preclinical testing and innovative approaches based on a deeper understanding of the pathophysiology of stroke.

## Figures and Tables

**Table 1:** Neuroprotective treatments currently investigated in phase II and phase III clinical trials.

Treatment	Mode of action
Magnesium Sulfate	Anti-excitotoxic, NMDA ion channel blocker
Albumin	Antioxidant, Hemodiluiting agent
Cyclosporin A	Anti-inflammatory, anti-excitotoxic
Dapsone (diamino-diphenyl sulfone, DDS)	Anti-inflammatory, antioxidant
Deferoxamine mesylate	Iron chelator, antioxidant
Ebselen	Antioxidant, free radical scavenger
GM602	Anti-apoptotic and anti-inflammatory
Hypothermia	Reduce cerebral oxygen metabolism, synaptic inhibitor
Lovastatin	Antioxidant, HMGCoA inhibitor
Minocycline	Anti-inflammatory, antioxidant
PG2 (Polysaccharides of Astragalus membranaceus)	Chinese Herb, assumed antioxidative and anti-inflammatory
Simvastatin	Antioxidant, HMGCoA inhibitor
Spheno-Palatine Ganglion (SPG) stimulation	Induction of cerebral vasodilatation
THR-18	Synthetic plasminogen activator inhibitor
Transcranial laser therapy	Mitochondrial stimulation

Information on ongoing clinical studies gathered from the databases Clinicaltrials.gov (August 2012) and Strokecenter.org (August 2012) [10,43].
